# Development and validation of a predictive model for side branch flow impairment following stent implantation in patients with non-left main coronary bifurcation lesions: a retrospective analysis

**DOI:** 10.1186/s12872-026-06069-0

**Published:** 2026-06-17

**Authors:** Linlin Wang, Haoran Zhang, Aoxue Mei, Shuang Xie, Xintong Han, Lanqi Zeng, Jiamei Liu, Yang Jiao, Ying Zhang

**Affiliations:** 1 Department of Cardiology, The Affiliated Hospital of Chengde Medical University, Chengde, 067000 China; 2 Hebei Key Laboratory of Panvascular Diseases, Chengde, 067000 China; 3 The Cardiovascular Research Institute of Chengde, Chengde, 067000 China

**Keywords:** Coronary artery disease, Percutaneous coronary intervention, Coronary bifurcation lesions, Side branch flow impairment, Predictive model

## Abstract

**Objectives:**

The purpose of this study was to develop and validate a predictive model to assessing the risk of side branch flow impairment (SBFI) following stent implantation in patients with non-left main coronary bifurcation lesions (CBLs). This model aims to provide preprocedural risk stratification and inform the selection of interventional strategies.

**Background:**

Coronary artery bifurcation lesions constitute a particularly complex subtype of coronary artery disease that is frequently encountered in practice. Compared with non-bifurcation lesions, they are associated with greater procedural complexity and risk of procedure-related complications.

**Patients and methods:**

Data from 830 patients with CBL who underwent percutaneous coronary intervention (PCI) in the Affiliated Hospital of Chengde Medical University from January 2022 to December 2023 were retrospectively collected. The least absolute shrinkage and selection operator regression methods were used to screen variables, and multivariate logistic regression was used to establish a predictive model. A nomogram was built based on these factors and internally verified using the bootstrap resampling method. The C-statistic was used to verify and evaluate the discriminative ability of the model; the calibration curve was drawn, and the decision curve analysis (DCA) was performed to evaluate the calibration degree, clinical net benefit, and practicability of the model. The primary endpoint was SBFI, defined as a transient or persistent reduction in thrombolysis in myocardial infarction (TIMI) flow grade in a branch vessel following stent implantation in a major non-left main coronary artery.

**Results:**

A nomogram was constructed using the selected predictors of SBFI, which included age, plaque location ipsilateral to the SB, TIMI flow grade before main vessel (MV) stenting, and N-terminal pro-brain natriuretic peptide (NT-proBNP). The discriminatory ability of the model, as assessed by the area under the curve (AUC), was 0.651. The robustness of the model was evaluated through internal validation with 1000 bootstrap replicates, resulting in a corrected AUC of 0.641. The calibration curve, evaluated by the Hosmer–Lemeshow test, showed good agreement between predictions and observations (χ^2^ = 5.765, *P* = 0.674). Finally, DCA indicated that the model provided clinical net benefit over a threshold probability range of 0.19–0.45.

**Conclusion:**

We developed and validated a predictive model for SBFI after PCI in non-left main bifurcation lesions. The model exhibited modest discriminative ability, calibration, and a positive net benefit on DCA, suggesting that it may serve as a valuable risk stratification tool in clinical practice.

**Supplementary Information:**

The online version contains supplementary material available at https://doi.org/10.1186/s12872-026-06069-0.

## Introduction

Coronary bifurcation lesions (CBLs) represent a complex and high-risk subset of coronary artery disease (CAD), accounting for 15–20% of all percutaneous coronary interventions (PCIs). These lesions are characterized by greater procedural complexity and an elevated risk of adverse outcomes, including restenosis and stent thrombosis, compared with non-bifurcation lesions [1, 2]. A landmark prospective study established bifurcation lesions as an independent predictor of stent thrombosis, confirming their persistent clinical significance in the contemporary drug-eluting stent era [3].

The pathophysiology of side branch (SB) occlusion involves both anatomical and procedural factors. Biomechanically, coronary bifurcations demonstrate characteristic flow disturbances with regions of low oscillatory wall shear stress that correspond to sites of early atherosclerotic development and create an environment that is prone to thrombosis [4, 5]. Procedurally, main vessel (MV) stenting can mechanically compromise the SB ostium through carina shift, plaque redistribution, or stent strut obstruction [2, 6]. The selection of stenting strategy—whether provisional single-stent or complex two-stent techniques such as DK-crush, mini-crush, or nano-crush—significantly impacts the risk of SB compromise [1, 7]. Despite its widespread use, the conventional approach of MV stenting with provisional SB intervention carries a 6.1–18% risk of SB occlusion [8]. Although infrequent, procedural complications can further exacerbate this risk [6].

Preserving SB patency remains a central challenge in bifurcation PCI. Current risk assessment tools are limited by their failure to incorporate key clinical variables, which restricts their universal applicability for diagnosing specific non-left main coronary artery branch lesions. To address this need, we developed a predictive model that integrates clinical and angiographic parameters to generate individualized risk assessments. This approach aims to enhance procedural planning and clinical decision-making, with the ultimate goal of improving patient outcomes.

## Material and methods

### Study population

**Fig. 1:**
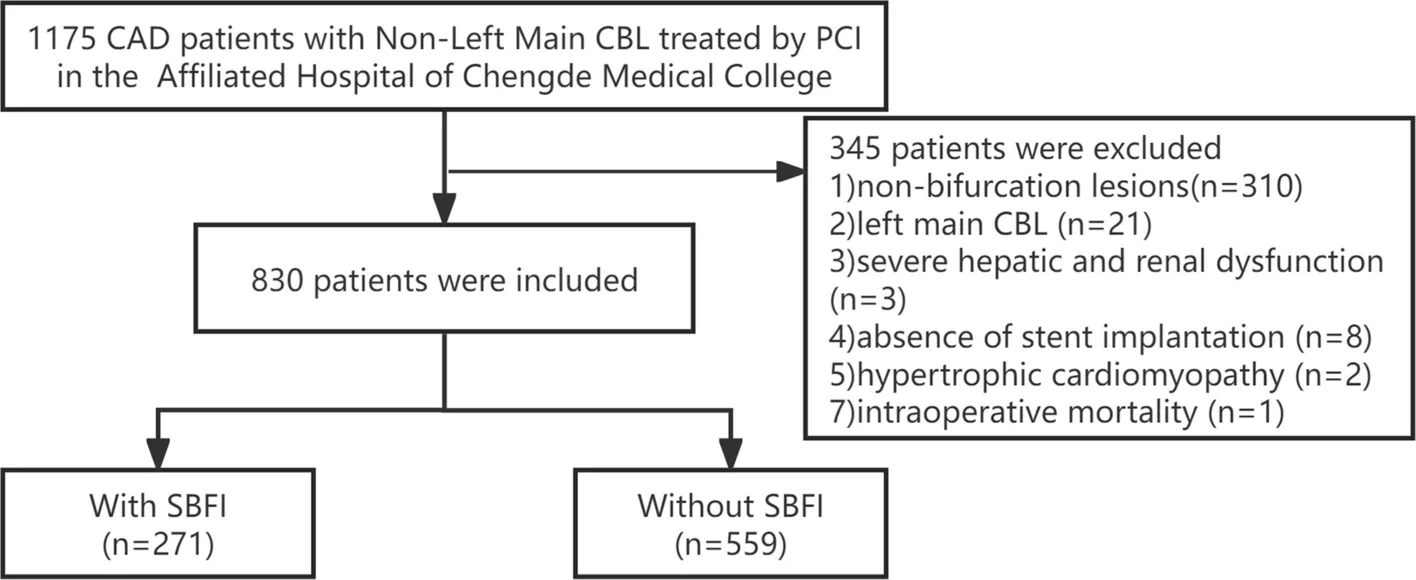
Patient selection flowchart

Patients with CAD admitted to the Affiliated Hospital of Chengde Medical University between January 2022 and December 2023 who had non-left main CBL and underwent PCI were selected. A total of 1175 patients who met the inclusion criteria were selected for this study. The inclusion criteria were as follows: (1) age ≥ 18 years; (2) fulfillment of the diagnostic criteria for CAD, according to the American College of Cardiology/American Heart Association (ACC/AHA) guidelines; (3) presence of a target lesion at a non-left main coronary artery (MCA) bifurcation, as defined by the European Bifurcation Club (EBC) criteria; (4) candidacy for PCI with provision of informed consent. The exclusion criteria were as follows: (1) stenting already performed at the ostium of the SB involving the target lesion; (2) presence of a chronic total occlusion (CTO) in either the MV or SB; (3) concomitant cardiac conditions such as congenital heart disease, pericardial disease, cardiomyopathy, or severe valvular heart disease; (4) severe dysfunction of major organs (e.g., hepatic or renal); (5) hematologic or coagulopathic disorders; and (6) cognitive impairment or psychiatric conditions. A flow diagram of the selection process is presented in Fig. 1.

This study was approved by the Ethics Committee of the Affiliated Hospital of Chengde Medical University (approval Number: CYFYLL2025057) and conducted according to the tenets of the Declaration of Helsinki. All the participants provided informed consent.

### Definitions

A CBL was defined as a stenosis adjacent to or involving the origin of a significant SB for which PCI was planned. In accordance with the recommendations of the EBC [9], a significant SB was one that the operator deemed essential to preserve based on the overall clinical context. Operationally, a branch was considered significant if it had a reference diameter ≥ 2.0 mm; however, other factors such as coronary anatomic variation, its role as a collateralizing vessel, size of the myocardial territory it supplied, and the patient's clinical symptoms were also integral to this determination. The bifurcation core referred to the 5-mm segment of the MV proximal to the carina. The bifurcation angle was defined as the angle between the distal MV and the SB. The primary study endpoint was SB flow impairment (SBFI), defined as a transient or persistent reduction in thrombolysis in myocardial infarction (TIMI) flow grade in the SB following stent implantation in a major non-left MCA.

### Clinical data collection

The following clinical characteristics were included for analysis: sex; age; body mass index (BMI); smoking index [number of cigarettes smoked per day/20] × [smoking duration in years]; hypertension; dyslipidemia; diabetes mellitus; diagnostic classification of CAD [unstable angina (UA), ST-segment elevation myocardial infarction (STEMI), non-ST-segment elevation myocardial infarction (NSTEMI), and other categories]; history of old myocardial infarction, PCI, coronary artery bypass grafting (CABG), or stroke; medication received, including dual antiplatelet therapy (DAPT) and the type of dual antiplatelet medication; white blood cell count (WBC), hemoglobin (HGB), and platelet count (PLT); low-density (LDL-C) and high-density (HDL-C) lipoprotein cholesterol, triglycerides (TG), and total cholesterol (TC); serum creatinine (sCr); uric acid (UA) and N-terminal pro-B-type natriuretic peptide (NT-proBNP) levels; peak cardiac troponin I (cTNI) levels; and left ventricular end-diastolic dimension (LVEDD) and left ventricular ejection fraction (LVEF).

### Angiographic data collection and quantitative coronary angiography

With the approval of the catheterization laboratory at Chengde Medical University, coronary angiography data and procedural information were retrospectively collected. All the angiographic images were independently reviewed by two experienced interventional cardiologists who were blinded to the bifurcation lesion status. Any discrepancies were resolved by a third senior cardiologist.

Quantitative coronary angiography (QCA) was performed using standard quantitative analyses and definitions [10]. Angiograms obtained at baseline and after pre-dilation were analyzed with a computer-based system dedicated to bifurcation analysis (CAAS workstation, version 8.5.5, Pie Medical Imaging, Maastricht, The Netherlands). We obtained quantitative angiographic measurements of the four segments of the bifurcation lesion—the proximal MV, distal MV, SB, and bifurcation core segments. We comprehensively assessed a range of angiographic and procedural variables, including coronary artery distribution, bifurcation lesion location and Medina classification, reference diameters of the MV (both proximal and distal) and SB, as well as the degree of stenosis and lesion length for both vessels. Additional parameters included the diameter of the implanted MV stent, SB orifice diameter, plaque distribution at the bifurcation, bifurcation angle, and characteristics of the bifurcation core (lesion length and stenosis). We also evaluated the calcification, tortuosity, and the presence of thrombus in both vessels; presence of MV dissection; preprocedural TIMI flow grade (MV and SB); intraprocedural TIMI flow grade (SB); and the performance of SB balloon angioplasty or wire protection.

A derived variable, the main vessel-to-side branch (MV/SB) diameter ratio ([reference diameter of proximal MV + reference diameter of distal MV] ⁄ 2 [reference diameter of SB]), was calculated from raw QCA measurements. It served as an anatomical indicator quantifying the relative risk of plaque shift from the MV to the SB ostium.

### Statistical analysis

The Kolmogorov–Smirnov test was employed to assess the normality of continuous variables, with normally and non-normally distributed variables reported as mean ± standard deviation and median with interquartile range, respectively. Non-normally distributed continuous variables were compared using the Mann–Whitney U-test. Categorical variables were expressed as counts (%) and compared using the χ^2^ test.

Feature selection is imperative for constructing reliable models. Our cohort comprised 830 patients, including 271 SBFI events, adhering to the 1:10 events-per-variable (EPV) principle, limits the number of variables to approximately 27. With 57 initial variables, we utilized the least absolute shrinkage and selection operator (LASSO) regression methods for variable screening to enhance model robustness. The tuning parameter (lambda) was optimized via tenfold cross-validation. We chose the lambda.1se value—the largest lambda within one standard error of the minimum deviance—to yield a parsimonious model without appreciably compromising performance. The variables identified by LASSO were subsequently incorporated into a binary logistic regression model to develop the final nomogram, with each feature represented by its odds ratio (OR), 95% confidence interval (95% CI), and *P*-value. It is important to note that the statistical inference (P-values and confidence intervals) from this subsequent logistic model is conditional on the variable selection step and may be subject to optimism. Therefore, the primary evaluation of our model focuses on its overall predictive performance (AUC, calibration, net benefit) rather than on the significance of individual predictors. Continuous variables were modeled using restricted cubic splines (RCS) to accommodate potential nonlinear associations.

The discriminatory performance of the model was evaluated using the area under the receiver operating characteristic curve (AUC). To adjust for overfitting, internal validation was performed with 1000 bootstrap resamples, yielding an optimism-corrected AUC; values closer to 1.0 indicate stronger discriminative ability. To contextualize the performance of our newly developed model, we also calculated the RESOLVE [11] and V-RESOLVE [8] scores for each patient and assessed their discriminative ability for SBFI using ROC analysis within our cohort. Calibration was assessed visually using calibration plots, in which closer alignment between the predicted probabilities and 45° reference line indicated better model fit. Internal validation was performed with 1000 bootstrap resamples to reduce the potential for overfitting. The Hosmer–Lemeshow (H–L) and unreliability tests were also conducted, both of which produced nonsignificant results (*P* > 0.05), indicating adequate calibration across risk strata. Decision curve analysis (DCA) was employed to assess the clinical utility of the predictive model by quantifying the net benefit across a range of clinically relevant probability thresholds. In the DCA plot, the x-axis indicates the threshold probability, while the y-axis represents the net benefit. The analysis includes two reference lines: a horizontal line denoting the "Treat None" strategy (assuming all samples are negative, net benefit = 0), and an oblique line representing the "Treat All" strategy (assuming all samples are positive). The clinical value of the model is demonstrated when its decision curve lies above both these reference lines, with the area between the model curve and these lines indicating the range of threshold probabilities in which the model provides net clinical benefit.

For clinical application, the model was presented as a nomogram. Each predictor was assigned a points scale based on its regression weight. The total points, derived from adding the individual scores, were converted into the corresponding outcome probability using the nomogram.

All statistical analyses were performed using SPSS (version 26, SPSS Inc., Chicago, IL, USA) and R version 4.3.3. The ‘caret’, ‘foreign’, ‘rms’, ‘glmnet, ‘pROC’, and ‘rmda’ packages, among others, were used for data collation and visualization.

## Results

### Patients’ characteristics

From an initial cohort of 1175 patients with CAD, 345 were excluded. Most of these patients (*n* = 310) were excluded as they had non-bifurcation lesions; other reasons for exclusion were left main CBL (*n* = 21), severe hepatic and renal dysfunction (*n* = 3), absence of stent implantation (*n* = 8), hypertrophic cardiomyopathy (*n* = 2), and intraoperative mortality (*n* = 1). Ultimately, 830 patients were included in the final analysis. Of these, 271 developed SBFI, yielding an SBFI incidence rate of 32.6%. A univariate analysis of 57 relevant variables for enrolled patients revealed that age, lesion length of bifurcation core, diameter stenosis of SB (range), plaque located at the same side of SB, TIMI flow grade before MV stenting, and SB pre-dilation were significantly associated with SBFI (*P* < 0.05). In contrast, the remaining variables did not reach statistical significance (*P* > 0.05) (Tables 1 and 2).

**Table 1:** Baseline clinical characteristics

Variables	Total (*n* = 830)	Non-SBFI group (*n* = 559)	SBFI group (*n* = 271)	χ2/Z	*P* -value
Demographic					
Male (n, %)	583 (70.2%)	397 (71.0%)	186 (68.6%)	0.597	0.481
Age (years)	59.95 ± 9.88	59.14 ± 9.76	61.62 ± 9.94	−3.410	< 0.001
Dyslipidemia (n, %)	219 (26.4%)	154 (27.5%)	65 (24.0%)	1.194	0.275
Hypertension (n, %)	535 (64.5%)	357 (63.9%)	178 (65.7%)	0.263	0.608
Diabetes mellitus (n, %)	244 (29.4%)	159 (28.4%)	85 (31.4%)	0.751	0.386
Previous stroke (n, %)	165 (19.9%)	103 (18.4%)	62 (22.9%)	2.272	0.132
Myocardial infarction in 1 month (n, %)	90 (10.8%)	55 (9.8%)	35 (12.9%)	1.786	0.181
Previous PCI (n, %)	100 (12.1%)	60 (10.7%)	40 (14.8%)	2.793	0.095
Previous CABG (n, %)	1 (0.1%)	1 (0.2%)	0 (0.0%)	0.485	0.486
BMI (kg/m^2^)	25.64 ± 6.15	25.47 ± 3.57	26.00 ± 9.46	−1.166	0.244
Smoking Index (pack-years)	100.00 (0.00, 600.00)	140.00 (0.00, 600.00)	50.00 (0.00, 600.00)	−0.676	0.499
Laboratory data					
WBC (10^9^/L)	8.08 ± 3.05	8.14 ± 3.12	7.95 ± 2.88	0.835	0.404
HGB (g/L)	143.31 ± 36.25	144.70 ± 42.47	140.44 ± 17.16	1.586	0.113
PLT (10^9^/L)	225.13 ± 58.65	225.96 ± 57.46	223.42 ± 61.09	0.586	0.558
TC (mmol/L)	4.33 ± 1.20	4.37 ± 1.26	4.24 ± 1.07	1.524	0.128
TG (mmol/L)	1.37 (0.98, 1.97)	1.33 (0.97, 2.01)	1.44 (1.04, 1.90)	−0.530	0.596
HDL-C (mmol/L)	1.12 ± 0.31	1.13 ± 0.32	1.12 ± 0.30	0.597	0.551
LDL-C (mmol/L)	2.70 ± 0.99	2.74 ± 1.02	2.62 ± 0.92	1.569	0.117
Cr (umol/L)	64.45 (54.93, 74.70)	64.40 (54.55, 74.55)	64.60 (55.90, 75.45)	−0.441	0.659
UA (umol/L)	317.39 (260.90, 374.87)	318.00 (260.62, 380.14)	315.24 (261.69, 369.67)	−0.806	0.420
NT-proBNP (pg/mL)	75.30 (20.00, 252.00)	68.10 (20.00, 238.50)	87.00 (20.00, 268.50)	−1.468	0.142
Positive-cTNI (ng/mL)	323 (38.9%)	225 (40.3%)	98 (36.2%)	1.283	0.257
LVEDD (mm)	50.31 ± 4.75	50.09 ± 4.72	50.75 ± 4.77	−1.880	0.060
LVEF (%)	57.02 ± 8.94	56.84 ± 9.01	57.39 ± 8.80	−0.821	0.412
Clinical classification (n, %)				3.446	0.328
UA	443 (53.4%)	287 (51.3%)	156 (57.6%)		
STEMI	237 (28.6%)	170 (30.4%)	67 (24.7%)		
Non-STEMI	92 (11.1%)	63 (11.3%)	29 (10.7%)		
Others	58 (7.00%)	39 (7.0%)	19 (7.0%)		
Drugs (n, %)					
Aspirin	792 (95.4%)	537 (96.1%)	255 (94.1%)	1.619	0.203
P2Y12	826 (99.5%)	557 (99.6%)	269 (99.3%)	0.043	0.836
Statins	827 (99.6%)	558 (99.8%)	269 (99.3%)	1.584	0.208

Data are presented as n (%) or as the median [range]

*Abbreviations*: *SBFI* Side branch flow impairment, *PCI* Percutaneous coronary intervention, *CABG* Coronary artery bypass grafting, *BMI* Body mass index, *WBC* White blood cell, *HGB* Hemoglobin, *PLT* Platelet, *TC* Total cholesterol, *TG* Triglyceride, *HDL‐C* High‐density lipoprotein cholesterol, *LDL‐C* Low density lipoprotein cholesterol, *Cr* Creatinine, *UA* Uric acid, *NT-proBNP* N-terminal pro-B-type natriuretic peptide, *cTNI* Cardiac troponin I, *LVEDD* Left ventricular end-diastolic dimension, *LVEF* Left ventricular ejection fraction, *UA* Unstable angina, *STEMI* ST-segment elevation myocardial infarction, *Non-STEMI* Non-ST-segment elevation myocardial infarction

**Table 2:** Baseline lesion characteristics and stenting techniques

Variables	Total (*n* = 830)	Non-SBFI group (n = 559)	SBFI group (*n* = 271)	χ2/Z	*P* -value
Coronary artery distribution (n, %)				5.039	0.080
Left dominant coronary	46 (5.5%)	29 (5.2%)	17 (6.3%)		
Right dominant coronary	720 (86.8%)	479 (85.7%)	241 (89.0%)		
Codominant coronary	64 (7.7%)	51 (9.1%)	13 (4.8%)		
Location of bifurcation (n, %)				1.990	0.370
LAD/diagonal	524 (63.1%)	345 (61.7%)	179 (66.1%)		
LCX/OM	176 (21.2%)	126 (22.5%)	50 (18.5%)		
RCA bifurcation	130 (15.7%)	88 (15.7%)	42 (15.5%)		
Medina (n, %)				12.278	0.056
1,0,0	77 (9.3%)	64 (11.4%)	13 (4.8%)		
0,1,0	123 (14.8%)	84 (15.0%)	39 (14.4%)		
1,1,0	103 (12.4%)	66 (11.8%)	37 (13.7%)		
1,1,1	352 (42.4%)	235 (42.0%)	117 (43.2%)		
0,0,1	5 (0.6%)	4 (0.7%)	1 (0.4%)		
1,0,1	37 (4.46%)	21 (3.8%)	16 (5.9%)		
0,1,1	133 (16.0%)	85 (15.2%)	48 (17.7%)		
Bifurcation angle(°)				2.728	0.256
< 70	346 (41.7%)	225 (40.3%)	121 (44.6%)		
70– < 90	331 (39.9%)	223 (39.9%)	108 (39.9%)		
≥ 90	153 (18.4%)	111 (19.9%)	42 (15.5%)		
Diameter stenosis of bifurcation core (%)				2.454	0.293
< 50	451 (54.3%)	294 (52.6%)	157 (57.9%)		
50– < 70	154 (18.6%)	105 (18.8%)	49 (18.1%)		
≥ 70	225 (27.1%)	160 (28.6%)	65 (24.0%)		
Diameter of MV stent (mm)	3.00 ± 0.42	3.00 ± 0.42	3.01 ± 0.42	−0.625	0.532
Diameter of bifurcation core (mm)	2.51 ± 1.15	2.47 ± 1.17	2.58 ± 1.10	−1.263	0.207
Lesion length of bifurcation core (mm)	2.60 (0.00, 5.00)	3.00 (0.50, 5.00)	2.00 (0.00, 4.00)	−2.256	0.024
Lesion length of MV (mm)	25.00 (19.00, 34.00)	25.00 (19.00, 35.00)	24.00 (18.50, 32,000)	−0.898	0.369
Lesion length of SB (mm)	5.00 (0.00, 18.00)	5.00 (0.00, 18.00)	6.00 (0.00, 17.00)	−0.936	0.349
Diameter of the proximal Part of the MV (mm)	2.50 (1.50, 3.50)	2.50 (1.45, 3.50)	2.75 (1.50, 3.50)	−0.663	0.507
Diameter of the distal End of the MV (mm)	1.00 (0.50, 2.00)	1.00 (0.50, 2.00)	1.250 (0.40, 2.00)	−1.097	0.273
Diameter of SB (mm)	2.00 (1.03, 2.20)	2.00 (1.00, 2.10)	2.00 (1.50, 2.25)	−1.435	0.151
The max angulation of MV (°)	25.00 (5.00, 85.00)	25.00 (2.00, 85.00)	30.00 (5.00, 86.00)	−1.117	0.264
Diameter stenosis of MV (%)				5.054	0.409
0	2 (0.2%)	2 (0.4%)	0 (0.0%)		
< 25	6 (0.7%)	5 (0.9%)	1 (0.4%)		
25–49	12 (1.4%)	7 (1.3%)	5 (1.8%)		
50–74	66 (8.0%)	42 (7.5%)	24 (8.9%)		
75–89	183 (22.0%)	115 (20.6%)	68 (25.1%)		
≥ 90	561 (67.6%)	388 (69.4%)	173 (63.8%)		
Diameter stenosis of SB (%)				11.258	0.046
0	266 (32.0%)	193 (34.5%)	73 (26.9%)		
< 25	42 (5.1%)	21 (3.8%)	21 (7.7%)		
25–49	78 (9.4%)	47 (8.4%)	31 (11.4%)		
50–74	155 (18.7%)	106 (19.0%)	49 (18.1%)		
75–89	134 (16.1%)	87 (15.6%)	47 (17.3%)		
≥ 90	155 (18.7%)	105 (18.8%)	50 (18.5%)		
Plaque located at the same side of SB (n, %)	519 (62.5%)	370 (66.2%)	149 (55.0%)	9.786	0.002
TIMI flow grade before MV stenting (n, %)				25.379	< 0.001
TIMI 3	136 (16.4%)	105 (18.8%)	31 (11.4%)		
TIMI 2	99 (12.0%)	74 (13.2%)	25 (9.2%)		
TIMI 1	202 (24.3%)	149 (26.7%)	53 (19.6%)		
TIMI 0	393 (47.3%)	231 (41.3%)	162 (59.8%)		
SB contain thrombus (n, %)	59 (7.1%)	41 (7.3%)	18 (6.6%)	0.133	0.716
SB predilation (n, %)	140 (16.9%)	84 (15.0%)	56 (20.7%)	4.137	0.042
Two-stent strategy (n, %)	84 (10.1%)	55 (9.8%)	29 (10.7%)	0.149	0.699
Protection of SB guidewire (n, %)	611 (73.6%)	404 (72.3%)	207 (76.4%)	1.589	0.208
MV present tortuosity (n, %)				0.259	0.967
None	490 (59.0%)	328 (58.7%)	162 (59.8%)		
Mild	226 (27.2%)	154 (27.5%)	72 (26.6%)		
Moderate	71 (8.6%)	47 (8.4%)	24 (8.9%)		
Severe	43 (5.2%)	30 (5.4%)	13 (4.8%)		
SB present tortuosity (n, %)				1.475	0.688
None	501 (60.4%)	332 (59.4%)	169 (62.4%)		
Mild	203 (24.4%)	137 (24.5%)	66 (24.4%)		
Moderate	78 (9.4%)	57 (10.2%)	21 (7.7%)		
Severe	48 (5.8%)	33 (5.9%)	15 (5.5%)		
Calcification at the proximal end of MV (n, %)	280 (33.7%)	191 (34.2%)	89 (32.9%)	0.144	0.705
Calcification at the distal end of MV (n, %)	151 (18.2%)	107 (19.1%)	44 (16.2%)	1.035	0.309
Calcification of SB (n, %)	73 (8.8%)	56 (10.0%)	17 (6.2%)	3.191	0.074
MV exhibit dissection (n, %)	6 (0.7%)	4 (0.7%)	2 (0.7%)	0.001	0.971
MV contain thrombus (n, %)	138 (16.6%)	91 (16.3%)	47 (17.3%)	0.149	0.699
Diameter ratio between MV/SB (n, %)				1.797	0.616
< 1.0	419 (50.5%)	281 (50.3%)	138 (50.9%)		
1.0- < 1.5	210 (25.3%)	137 (24.5%)	73 (26.9%)		
1.5- < 2.0	85 (10.2%)	57 (10.2%)	28 (10.3%)		
≥ 2.0	116 (14.0%)	84 (15.0%)	32 (11.8%)		
RESOLVE score	21.72 ± 9.17	21.30 ± 9.26	22.59 ± 8.95	−1.974	0.048
V-RESOLVE score	21.35 ± 9.05	20.90 ± 9.08	22.30 ± 8.93	−2.089	0.037

Data are presented as n (%) or as the median [range]. Two‑stent strategy includes both planned two‑stent techniques and bailout stenting. Bailout stenting was performed in 20 cases (2.4% of the total cohort), all of which occurred in the SBFI group

*Abbreviations*: *SBFI* Side branch flow impairment, *LAD* Left anterior descending coronary artery, *LCX* Left circumflex coronary artery, *OM* Obtuse marginal branch, *RCA* Right coronary artery, *MV* Main vessel, *SB* Side branch, *TIMI* Thrombolysis In Myocardial Infarction

**Fig. 2:**
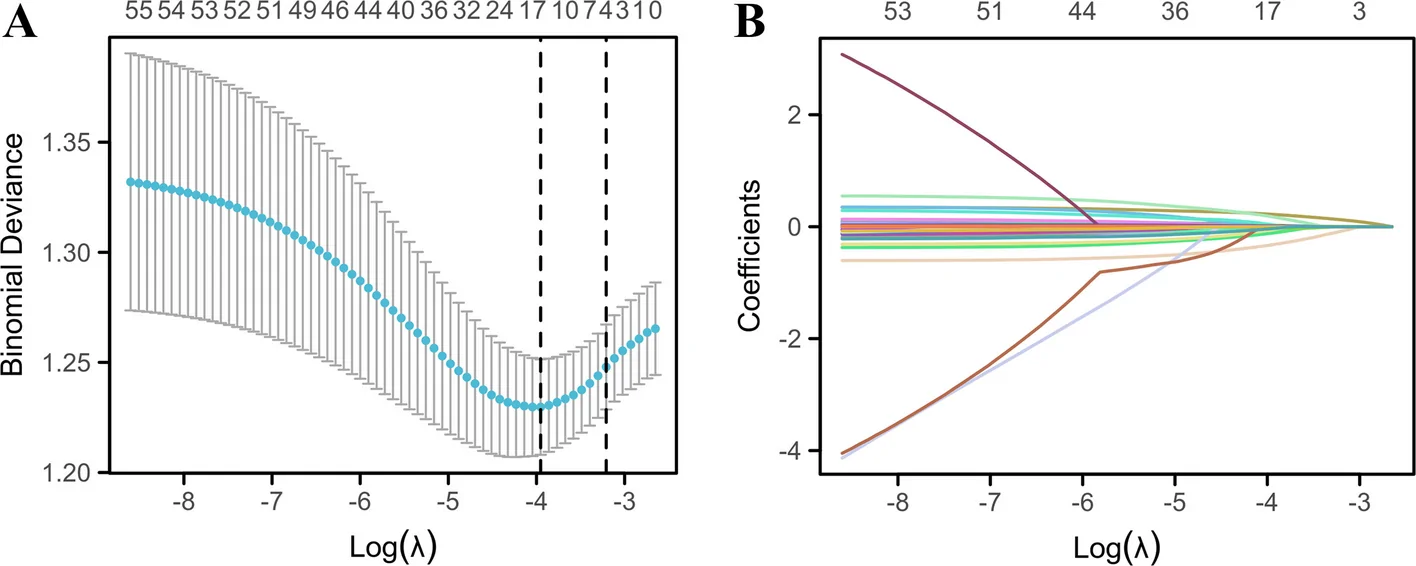
Texture feature selection via LASSO-logistic regression. **A** Selection of the optimal tuning parameter (λ) using tenfold cross-validation. The dotted vertical lines indicate the λ values for the minimum cross-validation error (left) and one-standard-error (1-SE criterion, right) rules. **B** The coefficient profile plot shows the trajectories of feature coefficients against the log (λ) sequence. The vertical line marks the final λ value chosen for the model

All candidate variables were included in the least absolute shrinkage and LASSO regression analyses to identify predictors for model development. The optimal penalty parameters were λmin = –2.56571 and λ1se = –1.35627 (Fig. 2).

**Fig. 3:**
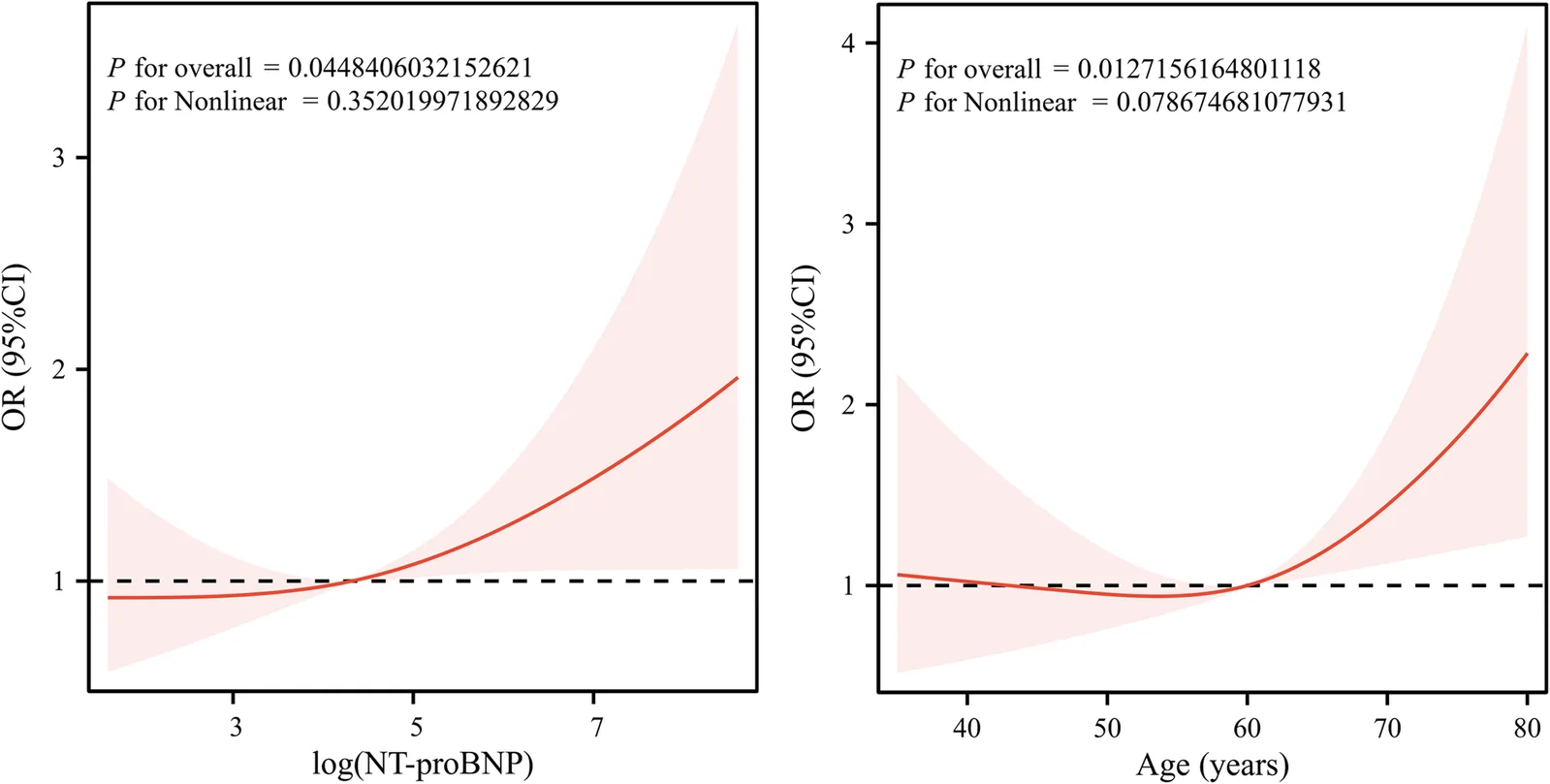
Restricted cubic spline (RCS)

Predictors retained in the final model were those with non-zero coefficients at λ1se, and included age, TIMI flow grade before MV stenting, plaque located at the same side as the SB, and NT-proBNP. Given its skewed distribution, NT-proBNP was log-transformed. Both log-transformed NT-proBNP and age were flexibly modeled with RCS using three knots (Fig. 3). Categorical variables—including plaque location and TIMI flow grade—were incorporated into the model as factors.

**Fig. 4:**
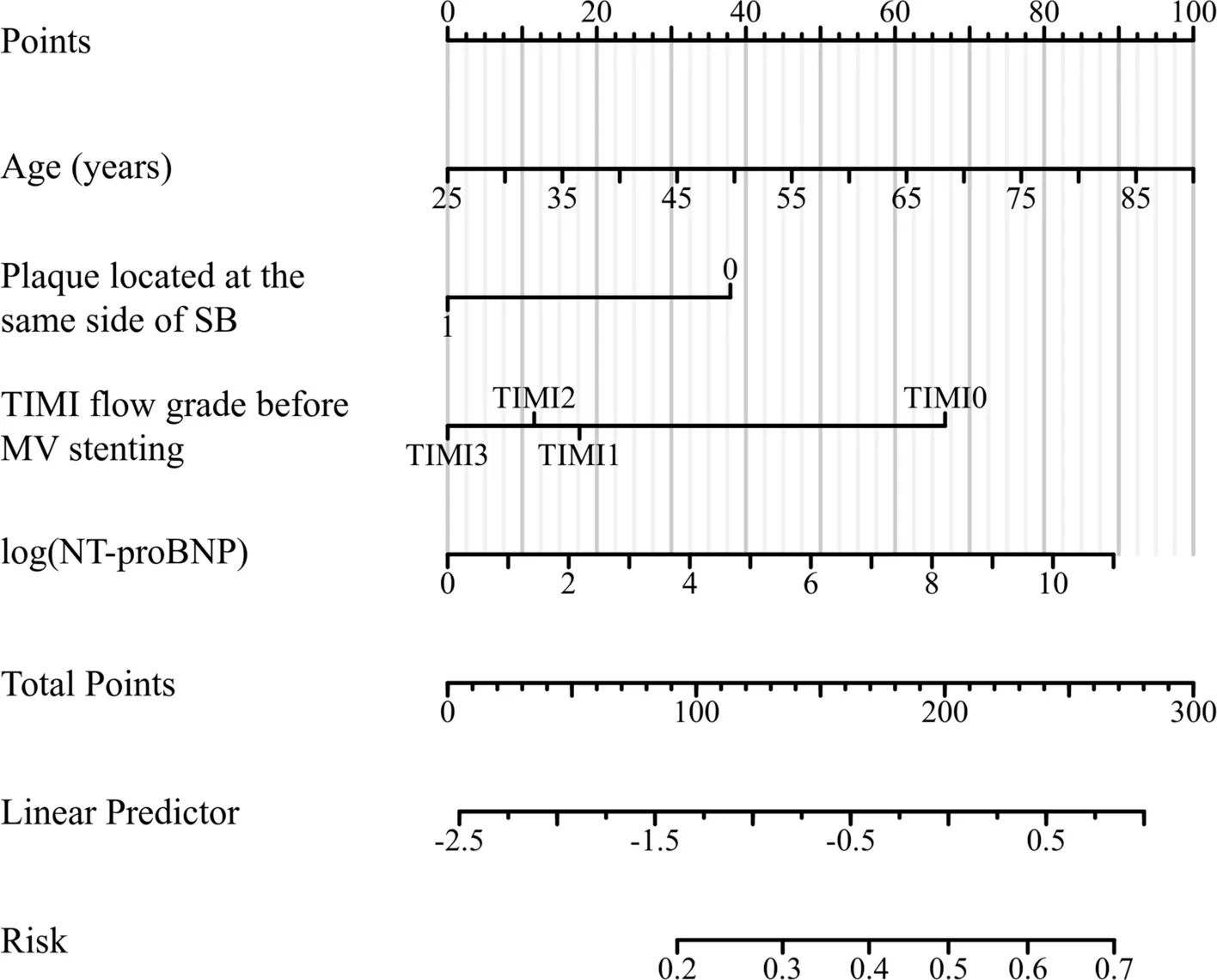
Nomogram predicting risk of SBFI

A multivariable logistic regression model was developed using the selected predictors. The final model results are summarized in Table 3 and included the following independent predictors: age (per year increase; OR: 1.020; 95% CI: 1.003–1.036; *P* = 0.019), plaque located at the same side as the SB (OR: 0.618; 95% CI: 0.453–0.842; *P* = 0.002), TIMI flow grade before MV stenting with TIMI grade 0 as reference (TIMI 1: OR: 0.536; 95% CI: 0.366–0.785; *P* = 0.001; TIMI 2: OR: 0.497; 95% CI: 0.297–0.830; *P* = 0.008; TIMI 3: OR: 0.428; 95% CI: 0.272–0.676; *P* < 0.001), and log-transformed NT-proBNP (OR: 1.109; 95% CI: 1.015–1.211; *P* = 0.022). Based on this model, a nomogram was constructed to predict the risk of SBFI after PCI in patients with non-left main CBL (Fig. 4).

**Table 3:** Results of multivariate logistic regression analysis

Variables	OR	95% CI	*P* -value
Age	1.020	1.003—1.036	0.019
Plaque located at the same side of SB	0.618	0.453—0.842	0.002
TIMI flow grade before MV stenting			
TIMI 0	1.000	Reference	
TIMI 1	0.536	0.366–0.785	0.001
TIMI 2	0.497	0.297–0.830	0.008
TIMI 3	0.428	0.272–0.676	< 0.001
Log (NT-proBNP)	1.109	1.015—1.211	0.022

*Abbreviations*: *OR* Odds ratio, *CI* Confidence interval, *SB* Side branch, *MV* Main vessel, *TIMI* Thrombolysis In Myocardial Infarction, *NT-proBNP* N-terminal pro-B-type natriuretic peptide

To use the nomogram, the value of each predictor is first aligned vertically with the "Points" axis to assign an individual score. The scores for all predictors are then added to obtain a total score. This total is subsequently projected downward onto the "Risk of SBFI" axis to determine the corresponding predicted probability of SBFI. For example, age contributes progressively from 0 to 100 points across the range of 25–90 years. TIMI flow grade before main vessel (MV) stenting, plaque located at the same side as the side branch (SB), and log-transformed NT-proBNP are each assigned specific scores based on their values. The overall SBFI risk is derived by adding all component scores and mapping the total to the risk axis.

### Interpretation of the nomogram model

**Fig. 5:**
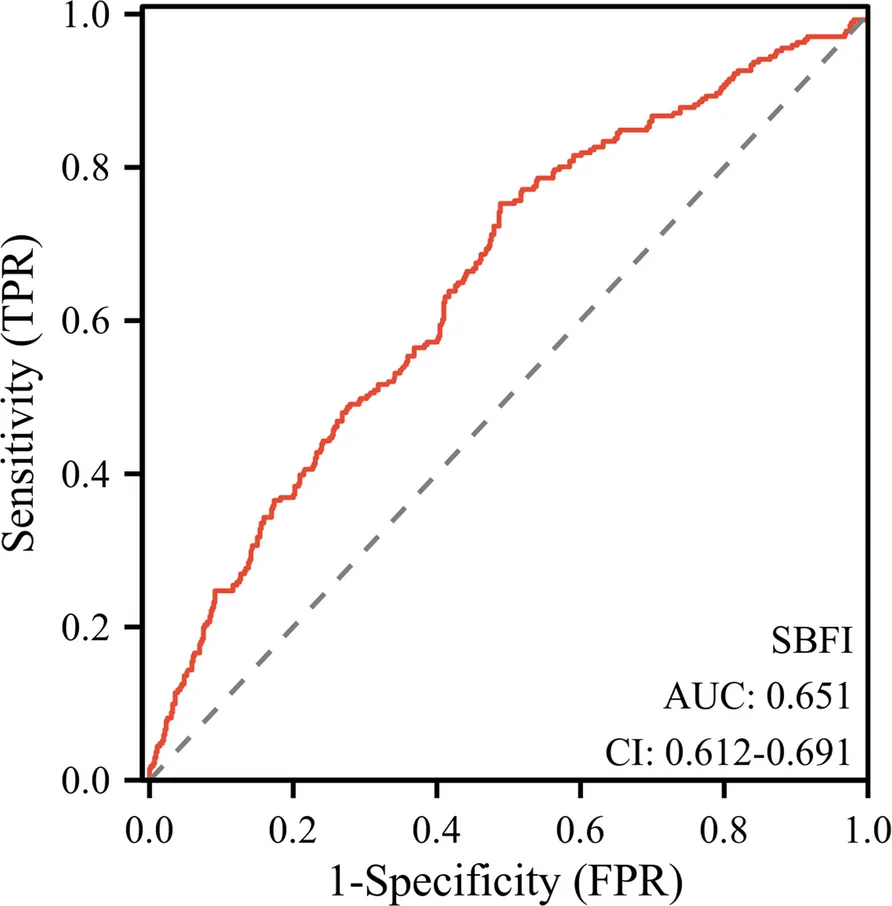
Receiver Operating Characteristic (ROC) curve

**Fig. 6:**
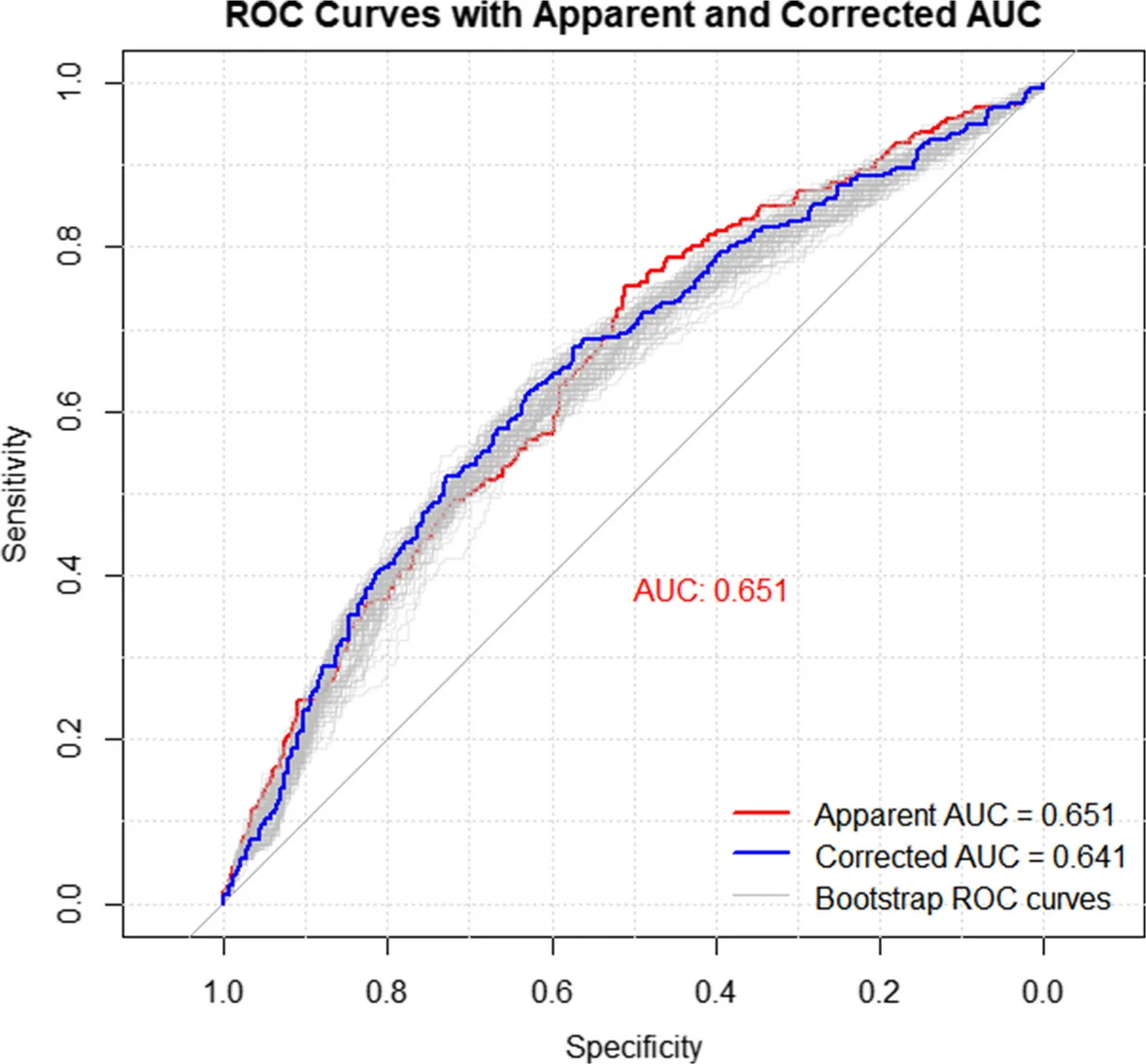
Bootstrap-validated ROC curve (1,000 replicates) showing the area under the curve (AUC) distribution (shaded area), original AUC (solid red line), and corrected AUC (solid blue line). Abbreviations: SBFI, side branch flow impairment

**Fig. 7:**
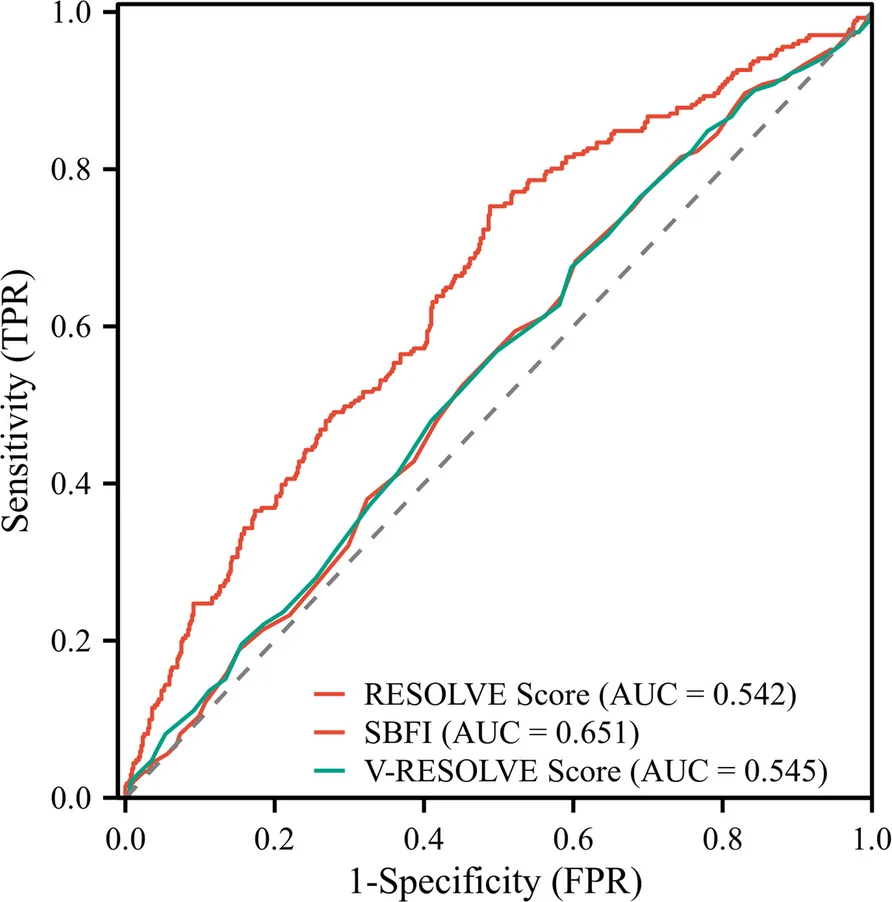
Comparison of ROC curves for SBFI prediction: novel model vs. established scores

As shown in Fig. 5, the model demonstrated an AUC of 0.651 (95% CI: 0.612–0.691). The discriminative performance was further characterized by an accuracy of 59.0%, sensitivity of 75.3%, and specificity of 51.2%. Internal validation using 1000 bootstrap resamples yielded a corrected AUC of 0.641, representing a minimal decrease of 0.01 from the original estimate and indicating well-maintained discriminatory performance (Fig. 6). For comparative purposes, the performance of two established angiographic scores was also assessed. The AUC for the RESOLVE score was 0.542, and for the V-RESOLVE score was 0.545 (Fig. 7).

**Fig. 8:**
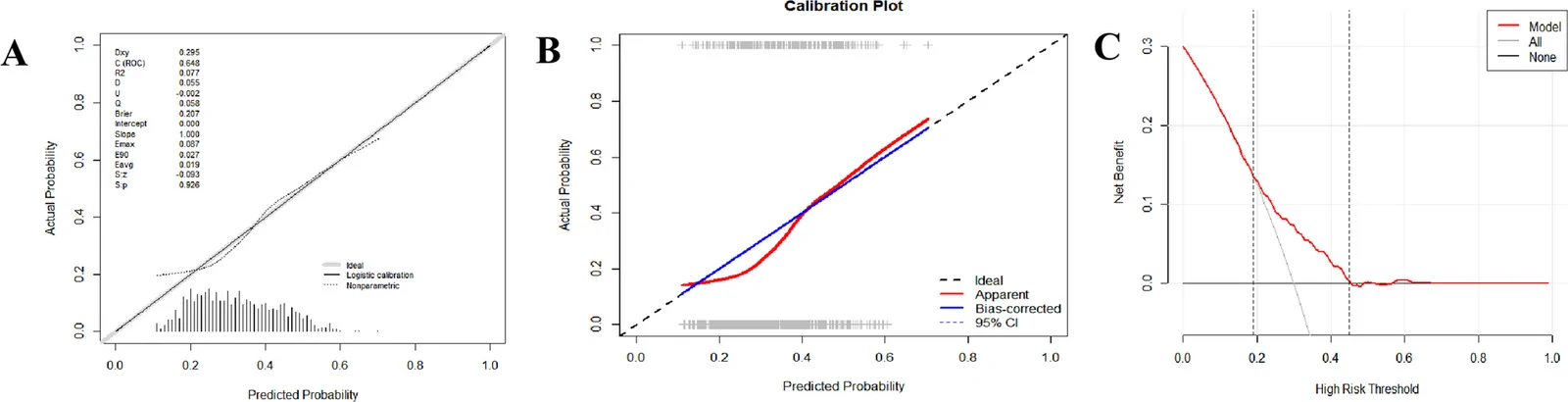
Calibration plot and Decision curve analysis (DCA) curve. **A** Calibration plot depicting the association between the nomogram-predicted and actual probabilities, showing the calibration curve of the original model. **B** Bootstrap-validated calibration curve (1,000 replicates) with the solid red and blue lines representing the original and bias-corrected calibration curves, respectively. **C** DCA curve

Calibration was assessed both graphically and statistically. The calibration plot demonstrated close agreement between the predicted probabilities and the observed outcomes. The unreliability test statistic U was –0.002 (*P* = 0.926), with maximum and average excursions of 0.087 and 0.019, respectively (Fig. 8A). Furthermore, the calibration and predictive consistency of the model were confirmed via bootstrap resampling (1000 iterations), as shown by the close alignment of the calibration curve in Fig. 8B. The Hosmer–Lemeshow test yielded a χ^2^ statistic of 5.765 (*P* = 0.674), collectively supporting good model calibration. DCA demonstrated that the model provided net clinical benefit within a threshold probability range of 0.19–0.45 (Fig. 8C).

## Discussion

The main findings of this study can be summarized as follows: (1) A novel predictive model incorporating two angiographic characteristics and two clinical variables was developed; (2) The model effectively predicts the risk of SBFI in patients undergoing PCI for non-left main CBL, demonstrating acceptable discriminative ability and good calibration upon internal validation.

CBLs account for 15–20% of all PCI procedures and represent a major challenge in interventional cardiology due to their anatomical complexity and technical demands [9, 12–14]. Compared with non-bifurcation interventions, bifurcation PCI is associated with higher procedural difficulty, lower immediate success rates, and increased risks of both acute and long-term complications [12, 14, 15]. Among the spectrum of potential complications, SBFI—particularly in its most severe form, side branch occlusion (SBO)—remains one of the most frequent and clinically consequential intraprocedural events [6]. The underlying mechanisms are multifactorial and well established, including plaque shift (“snowplow” effect), carinal displacement, and stent strut obstruction of the SB ostium [12, 14, 16]. Even transient impairment of SB perfusion can result in periprocedural myocardial injury or infarction, adversely affecting short- and long-term outcomes and procedural safety [6].

Given the clinical significance of SBFI, accurate risk stratification is crucial for procedural planning [9, 17]. Established angiographic scores such as RESOLVE and V-RESOLVE primarily incorporate anatomical factors (e.g., bifurcation angle, SB stenosis) but demonstrate only modest discriminative ability in our cohort (AUCs 0.542 and 0.545, respectively) [11, 16, 18–21]. Our model, which integrates a clinical biomarker (NT-proBNP) with angiographic variables (age, ipsilateral plaque, and preprocedural TIMI flow), showed improved discrimination (corrected AUC 0.641), along with good calibration and a positive net benefit across threshold probabilities of 0.19–0.45. This suggests added value in combining clinical and angiographic data for SBFI risk assessment.

Age is a key determinant of CAD complexity, especially in bifurcation lesions undergoing PCI. Older age correlates with greater plaque burden, reduced vascular compliance, and more complex anatomy, raising the risk of SB compromise. Our previous work suggests "vascular age" better reflects atherosclerotic burden than chronological age [22]. Elderly patients often present with calcified, multivessel bifurcation lesions and more comorbidities, increasing procedural risk [23–26]. Similarly, Kang et al. found that clinical factors (e.g., age, renal impairment) predict mortality, while angiographic features correlate with revascularization outcomes [27]. Thus, bifurcation PCI risk reflects not just age, but the confluence of physiological aging, comorbidities, and lesion morphology.

The hemodynamic environment at coronary bifurcations critically influences atherosclerotic plaque development, where flow patterns such as low wall shear stress and reverse flow promote platelet aggregation and lipid deposition [28, 29]. Numerical simulations indicate that larger bifurcation angles accentuate reverse flow near the side branch outer wall, fostering a plaque‑prone microenvironment [28]. During main vessel stenting, the "snowplow effect" may further alter local anatomy. The interplay of anatomy, hemodynamics, and pathology leads to asymmetric plaque distribution at bifurcation sites. While previous studies have identified ipsilateral plaque as a risk factor for branch occlusion [20], our analysis paradoxically suggests an association with reduced SBFI risk. This counterintuitive finding warrants cautious interpretation and should not be viewed as causal. We propose several explanatory hypotheses while acknowledging the limitations of our retrospective design. First, clearly visible ipsilateral plaque may have acted as a visual cue, prompting more frequent use of protective strategies—such as systematic SB wiring, pre‑dilation, or a lower threshold for two‑stent techniques—which could mitigate SBFI risk, though this procedural response was not directly captured [30, 31]. Second, unmeasured anatomical or procedural variables may confound the observed association. Third, the angiographic definition of “ipsilateral plaque” involves inherent subjectivity, and misclassification could bias results. Finally, given the modest effect size and multiple comparisons, this finding may represent a type I error or a cohort‑specific phenomenon. Thus, the apparent protective association likely reflects a complex interplay of procedural adaptation, unmeasured confounding, and cohort‑specific factors rather than a direct biological mechanism. This finding underscores the need for prospective studies that systematically document procedural decision‑making to disentangle plaque‑related risk from operator‑driven mitigation strategies.

The TIMI flow grade is a critical metric for assessing coronary perfusion and provides essential insight into the preprocedural hemodynamic status of the infarct-related artery [32]. A TIMI flow grade of 0 typically reflects a high thrombotic burden or unstable plaque pathology. Under these conditions, the presence of large thrombus or vulnerable plaque elevates the risk of distal embolization or "snowplow effect" during stent deployment. Additionally, impaired preprocedural flow may indicate underlying microvascular dysfunction, predisposing patients to no-reflow or slow-flow phenomena after stenting, which can subsequently compromise SB perfusion. Consistent with prior studies, our analysis incorporated preprocedural TIMI flow as a predictor and confirmed that impaired TIMI flow is associated with a significantly elevated risk of SBFI.

NT-proBNP is a sensitive biomarker reflecting myocardial wall stress and hemodynamic burden, with elevated levels observed in conditions such as heart failure, myocardial ischemia, renal dysfunction, and systemic inflammation [33]. It serves as an indicator of overall cardiovascular vulnerability and is a strong prognostic marker in ACS and other cardiovascular diseases. [34, 35]. Data from the PARADISE-MI trial demonstrate that elevated NT-proBNP during the early convalescent phase after high-risk myocardial infarction is associated with adverse cardiovascular outcomes [36]. In conditions such as myocardial infarction with non-obstructive coronary arteries (MINOCA), combining NT-proBNP with inflammatory markers improves risk stratification [37]. A substantial proportion of patients in our study presented with ACS, including STEMI, NSTEMI, and UA. In these patients, elevated NT-proBNP likely reflects the combined effects of acute ischemic injury, microcirculatory disturbance, and neurohormonal activation [38, 39]. These mechanisms may increase the vulnerability of side branch flow during procedural manipulation and reduce cardiac tolerance to flow interruption during PCI. Furthermore, impaired renal function reduces NT-proBNP clearance, leading to elevated baseline levels independent of cardiac stress. Studies in chronic kidney disease (CKD) populations, such as the GCKD study, confirm that NT-proBNP remains a reliable predictor of cardiovascular events in this setting [40]. Even in compensated CKD, elevated NT-proBNP may indicate subclinical myocardial strain and microvascular dysfunction [41]. Therefore, in patients with renal impairment or ACS, the predictive threshold for NT-proBNP may require adjustment, or its value may be enhanced through integration with other biomarkers, such as troponin and inflammatory markers, to improve the model’s applicability and accuracy across diverse clinical scenarios.

Current guidelines advocate a provisional single-stent strategy for most non‑left main bifurcation lesions [2, 42, 43]. For lesions with high angiographic complexity, a larger side‑branch diameter, or significant ostial involvement, more complex two‑stent techniques—such as DK crush, culotte, or T stenting—are selectively used [43, 44]. The choice of stenting strategy directly influences side‑branch outcomes: dual‑stent techniques inherently involve more extensive manipulation at the carina, potentially raising the risk of stent malapposition or strut‑induced obstruction. In contrast, the provisional approach aims to minimize side‑branch disturbance but remains vulnerable to plaque shift or carina displacement during MV stenting [45–47]. Recent consensus statements underscore that stent strategy should be individualized based on lesion morphology, functional significance of the side branch, and operator experience [2, 42, 48]. Furthermore, intravascular imaging (IVUS or OCT) plays an increasingly recognized role in optimizing stent sizing, expansion, and side branch accessibility, contributing to improved procedural outcomes [42, 48]. For CBLs with complex morphology and high thrombotic burden, an intensified peri‑procedural strategy is warranted, which may include mechanical adjuncts (e.g., thrombus aspiration) and optimized antithrombotic therapy. Beyond standard DAPT, the use of fast‑acting intravenous antiplatelet agents such as cangrelor—which offers immediate, reversible platelet inhibition—is particularly relevant and has been shown to reduce periprocedural ischemic events in high‑risk PCI, including thrombus‑rich bifurcation lesions [4, 49]. Post‑procedurally, tailored antithrombotic regimens based on individual ischemic‑bleeding risk profiles are crucial, with attention to DAPT duration and the potential need for intensified strategies in complex bifurcation interventions [4, 50]. Integrative risk models like ours could help identify patients most likely to benefit from such tailored approaches. Future studies should examine whether incorporating this model into clinical algorithms improves net clinical outcomes.

In clinical practice, this model can assist interventional cardiologists in stratifying patient risk prior to bifurcation PCI. The decision curve analysis indicated a net clinical benefit for the model across a threshold probability range of 0.19 to 0.45. Based on this, we propose the following risk-adapted management implications: a) For patients with a nomogram-predicted SBFI risk > 20% (i.e., above the lower bound of the beneficial range), we recommend mandatory side branch (SB) wire protection and a low threshold for SB pre-dilation or ballooning; b) For patients with a predicted risk > 35‑40% (approaching the high‑risk upper bound), a more intensified, proactive strategy is justified. This should include strong consideration of intravascular imaging (IVUS/OCT) guidance to optimize stent deployment and plaque assessment, and may warrant intensified periprocedural antithrombotic therapy (e.g., use of a potent intravenous P2Y12 inhibitor like cangrelor in selected high-thrombotic-risk cases). For instance, an older patient with TIMI 0 flow and markedly elevated NT-proBNP would score highly on the nomogram, yielding a predicted SBFI risk likely exceeding 40%. For this patient, the model directly supports the intensified strategy outlined above. Conversely, a younger patient with TIMI 3 flow and normal NT-proBNP would have a low predicted risk (< 20%), supporting a standard provisional stenting approach without mandatory complex protective measures. These scenarios underscore the model's utility in fostering individualized, risk-adapted procedural planning.

Its integration of NT-proBNP, a marker of myocardial stress and microvascular dysfunction, may provide complementary pathophysiological insight beyond purely anatomical scores, particularly in patients presenting with ACS or comorbid heart failure (HF). Therefore, the model's clinical utility lies in facilitating early identification of patients at elevated risk, thereby supporting more informed procedural planning and personalized treatment approaches. In practice, the model may serve as a pragmatic, integrative checklist that enriches preprocedural risk–benefit assessment, particularly in settings where more complex angiographic scoring systems are not routinely utilized.

### Study limitations

Several limitations of this study should be considered when interpreting its findings. First, its retrospective, single-center design carries inherent risks of selection bias and unmeasured confounding. Reliance on electronic medical records may have led to misclassification or missing data for some variables. Future prospective, multicenter studies are warranted for validation, and the establishment of standardized data collection platforms would help mitigate these issues. Second, although statistically significant, the discriminative ability of the model was modest (AUC = 0.651), indicating that a considerable portion of the variance in SBFI risk remains unexplained. To enhance predictive performance, future studies could incorporate a wider array of variables and explore machine learning techniques. Third, the absence of external validation restricts the generalizability of our findings. Independent validation in cohorts from diverse geographic, ethnic, and healthcare settings is needed to confirm robustness and applicability. Fourth, the modeling approach is subject to overfitting and post-selection inference limitations. Using LASSO regression for variable selection on the same dataset employed for final modeling may lead to optimistic effect estimates. Thus, the primary strength of the model lies in its overall predictive performance and clinical utility rather than in the statistical significance of individual predictors. Fifth, the primary endpoint of SBFI, defined by angiographic TIMI flow reduction, is susceptible to inter-observer variability and may be influenced by imaging acquisition or transient phenomena such as vasospasm. Although a blinded core lab adjudication process was used, measurement noise may attenuate the model's observed discriminatory performance. Sixth, as a preprocedural tool, our model does not incorporate intraprocedural variables—such as specific stenting strategy, intravascular imaging use, or operator experience—that significantly influence final outcomes. The primary objective of this study is to evaluate baseline anatomical and clinical risk rather than to predict results of specific procedural decisions, which may partly explain the model's modest discriminative performance. Therefore, the model should be interpreted primarily as a pre‑procedural risk‑stratification instrument. Future prospective studies that integrate these procedural factors are necessary to develop a more comprehensive and clinically actionable prediction model.

## Conclusion

In this study, we developed and validated a preprocedural risk stratification model for SBFI following PCI in patients with non-left main CBL. The model, which integrates four readily available clinical and angiographic variables—age, TIMI flow grade before MV stenting, plaque located at the same side, and NT-proBNP levels—demonstrated statistically modest discriminative ability (AUC = 0.651), good calibration, and provided a positive net benefit across a range of clinically relevant risk thresholds. By facilitating preprocedural risk stratification, this nomogram aids clinicians in tailoring their stenting strategy and peri-procedural planning, potentially improving the safety of bifurcation PCI. However, given the single-center, retrospective nature of this study and the lack of external validation, its broader applicability and clinical integration awaits confirmation through external validation.

## Supplementary Information

Additional file 1.

## Data Availability

The dataset(s) supporting the conclusions of this article is(are) included within the article (and its additional file(s)).

## Abbreviations

ACS: Acute coronary syndromes
AUC: Area under the curve
CAD: Coronary heart disease
CBL: Coronary bifurcation lesions
CI: Confidence interval
DCA: Decision curve analysis
EBC: European bifurcation club
H-L: Hosmer-Lemeshow test
LAD: Left anterior descending coronary artery
LCX: Left circumflex coronary artery
MCA: Main coronary artery
MV: Main vessel
Non-STEMI: Non-ST-segment elevation myocardial infarction
OM: Obtuse marginal branch
OR: Odds ratio
PCI: Percutaneous coronary intervention
QCA: Quantitative coronary angiography
RCA: Right coronary artery
RCS: Restricted cubic spline
ROC: Receiver operating characteristic
SB: Side branch
SBO: Side branch occlusion
SBFI: Side branch flow impairment
STEMI: ST-segment elevation myocardial infarction
TIMI: Thrombolysis in Myocardial Infarction
UA: Unstable angina
